# “I Just Really Felt Like I Was Heard for the First Time”: Survivor Experiences With an Adult Forensic Interviewing Model

**DOI:** 10.1177/10778012251379426

**Published:** 2025-09-22

**Authors:** Morgan E. PettyJohn, Aly Kramer Jacobs, Khara Breeden, Bethany L. Backes, Leila Wood

**Affiliations:** 1 School of Social Work, 12329 The University of Texas at Arlington, TX, USA; 2 Department of Psychiatry and Behavioral Sciences and Center for Violence Prevention, 12340 The University of Texas Health Science Center at Houston (UTHealth), TX, USA; 3 Graduate College of Social Work, University of Houston, TX, USA; 4 647651 Texas Forensic Nurse Examiners: Forensic Center of Excellence, TX, USA; 5 Department of Criminal Justice and School of Social Work, 6243 University of Central Florida, FL, USA

**Keywords:** adult forensic interviewing, intimate partner violence, sexual assault, criminal legal system, survivor services

## Abstract

Adult forensic interviewing (AFI) collects evidence for the investigation and prosecution of interpersonal violence in a trauma-informed setting. The present study uses mixed methods evaluation data from an AFI program in the U.S. to assess survivors’ perceptions of safety and the utility of participating in these interviews. Most participants felt the process was beneficial to their personal lives and criminal justice system engagement by: (a) allowing them to share their stories in more detail, (b) supporting their healing journeys, and (c) helping them feel safer through resource referral. AFI is a promising practice that warrants continued research.

## Introduction

Experiences of interpersonal violence (e.g., intimate partner violence, sexual assault, human trafficking) remain prevalent, with devastating consequences for individual survivors, their families, and society at large. Interpersonal violence refers to violence or abuse perpetrated against another person, including by family members, intimate partners, or acquaintances (World Health Organization, 2025). Estimates suggest nearly half of women (47%) and 44% of men are victims of intimate partner violence (IPV) in their lifetime, and over a quarter of women (27%) report lifetime experiences of attempted or completed rape (Basile et al., 2022; Leemis et al., 2022). Additionally, it is estimated that over one million people are currently living in conditions of human trafficking in the United States (Walk Free, 2023). These types of violence are even more common among individuals with marginalized racial, gender, and sexual identities (Basile et al., 2022; Chen et al., 2023; Leemis et al., 2022). Interpersonal violence is associated with a litany of negative outcomes for survivors, including mental and physical health consequences, financial insecurity, unstable housing, and substance misuse (Mason & O’Rinn, 2014; Stubbs & Szoeke, 2022; Yakubovich et al., 2022). Despite the prevalence rates and associated harms, crimes of interpersonal violence are drastically underreported and only a small proportion result in prosecution within criminal legal systems (Lonsway & Archambault, 2012; Thompson & Tapp, 2022).

Survivors may be reluctant to report to authorities for several reasons, including concerns for the perpetrator, fear for their safety, and distrust of law enforcement (Du Mont et al., 2003; Jones et al., 2009). For those who do report, it is crucial that they have an experience that enables and supports them to remain involved in a potential criminal or civil case, if they choose. Indeed, survivor disengagement due to safety concerns and/or negative interactions with law enforcement during investigations is a leading reason for sexual assault, IPV, and human trafficking cases failing to reach arrest or prosecution (Long & Garvey, 2012; Markey et al., 2021; Sleath & Smith, 2017). Survivors are often forced to recount their trauma numerous times to different people across medical, criminal, and legal systems. During these processes, many survivors report experiencing secondary victimization, or mistreatment by service providers, which extends and compounds the traumatic impacts of the original assault (Campbell & Raja, 1999; McQueen et al., 2021). Research and practice innovations over recent decades have aimed to improve these processes in the hopes of increasing survivor engagement, and therefore rates of successful prosecution, with one such model being adult forensic interviewing.

Adult forensic interviewing (AFI) is an emerging practice that involves collecting thorough evidence for legal prosecution of violent crimes in a setting designed to reduce burden and secondary victimization for survivors (Hunley & O’Donohue, 2023). AFIs are modeled after forensic interviews through child advocacy centers (CACs), utilizing specially trained interviewers as part of a multidisciplinary investigatory process. While a general outline of the approach has been established, literature is lacking and inconsistent regarding protocols and outcomes (Hunley & O’Donohue, 2023). Notably, only one study has examined the acceptability of AFIs among survivor participants (Kelly & Valentine, 2018). The current study adds to the growing knowledge base by exploring participant perceptions of AFIs, with a focus on safety and utility. Gaining a better understanding of AFI processes and participant experiences may help survivors access needed services and increase and maintain engagement in the criminal legal system among survivors who wish to access these resources.

## Reporting, Investigating, and Prosecuting Interpersonal Violence

In the United States, about a third (34%) of rapes/sexual assaults and just over half (58%) of intimate partner violence victimizations are reported to law enforcement (Morgan & Truman, 2020). Comparable crime victimization data are not available for human trafficking; however, over 2,000 alleged traffickers were referred to U.S. attorneys for prosecution in 2021 (USDOJ, 2023). Reports being filed with authorities are only the first step in an often-lengthy process of investigation and potential prosecution, both of which typically require active participation from survivors to be successful (Patterson & Campbell, 2010). Crimes of interpersonal violence are notoriously characterized by a “funnel of attrition,” whereby each stage of the process sees fewer and fewer cases progress toward incarceration or rehabilitation of perpetrators (Garner & Maxwell, 2009; Lonsway & Archambault, 2012). Case attrition occurs for many reasons, including survivor disengagement (Chenier et al., 2021), no arrests made by law enforcement (Addington & Rennison, 2008), district attorneys declining to prosecute the case (Spohn & Tellis, 2019), and judges providing lenient sentencing (Brown, 2012).

Composite crime victimization statistics suggest that, out of the one in three sexual assaults that are reported to law enforcement, roughly 16% result in an arrest, 9% result in a felony conviction, and 8% result in jail time (RAINN, 2024). The United States has seen significant increases in prosecution of IPV since the passage of the Violence Against Women Act in 1994, which raised awareness about gender-based violence, helped fund resources to address the issue, and ushered in new criminal justice policies (such as mandatory arrest); however, rates remain proportionately low (about one in four reported cases resulting in prosecution) (Garner & Maxwell, 2009). Prosecution of human trafficking cases has more than doubled across the United States (729–1,672) in the last decade (USDOJ, 2023). These statistics and efforts to increase the prosecution of crimes of interpersonal violence highlight the importance of identifying ways to best support survivors through the criminal justice process.

### Barriers to Survivor Engagement in Interpersonal Violence Investigations

In cases of interpersonal violence, pursuing arrest, prosecution, and sentencing of perpetrators requires a strong evidence base established during investigations. Despite advancements in forensic evidence over time (e.g., DNA collection) and a push towards evidence-based prosecution (Messing, 2014), law enforcement still predominantly relies on non-biological evidence for sex crime investigations, for a variety of reasons. For example, cases may not yield DNA, or the DNA may not be of high enough quality to upload into a database (Waltke et al., 2018). Moreover, the presence of DNA in rape kits is not inherently probative in assaults committed by known offenders who claim the contact was consensual (Menaker et al., 2017). Victim statements, credibility, and sustained involvement in cases are therefore paramount (Menaker et al., 2017; Waltke et al., 2018). However, cases of interpersonal violence lose survivor participation for several personal reasons (e.g., fear of retaliation, self-blame, compounding life stressors). Indeed, safety concerns loom large for survivors contemplating engagement with the legal system, discouraging some survivors from participating in investigations or pursuing legal action (Carey & Solomon, 2014; Long & Garvey, 2012). The need for financial and housing stability can deter participation, particularly in situations where children are involved and the perpetrator is a primary source of income (Yakubovich et al., 2022). Moreover, particularly in IPV cases, survivors generally share friends, family, and cultural communities with the perpetrator and risk severing access to these supports if they pursue charges. This concern is especially salient for survivors in marginalized communities which are distrustful of law enforcement (Holliday et al., 2020).

*Secondary Victimization and Institutional Betrayal*. In addition to personal barriers, survivors often experience secondary victimization and institutional betrayal from criminal legal systems, both of which can exacerbate trauma symptoms and may discourage continued participation with their case (Campbell et al., 2001; Smith & Freyd, 2013). Survivors can experience secondary victimization when they are blamed, doubted, or treated with suspicion for their assault at various stages of reporting, investigation, and prosecution processes (Orth, 2002; Patterson, 2012; Quinlan, 2016). People within law enforcement and the court systems are not immune to stereotypes and myths about interpersonal violence while processing these cases (Brown, 2012; Chenier et al., 2021), which can lead to this type of mistreatment. Moreover, survivor experiences can be worsened by adversarial procedures built into the criminal legal system, such as working to identify inconsistencies in survivor accounts and initially presuming the accused perpetrator is innocent (Parsons & Bergin, 2010). Institutional betrayal refers to failures of systems to protect constituents or respond appropriately after harm has been done (Smith & Freyd, 2013). Examples of this in the criminal legal system include delayed or inadequate responses from police (e.g., declining to arrest perpetrators; Gillis et al., 2006), poor investigation practices and loss of evidence (e.g., backlogs of untested rape kits; Campbell et al., 2024), prosecutors declining to file charges (Spears & Spohn, 1996), and judges declining protective orders or providing lenient sentencing (Muller et al., 2009; Smith et al., 2014), among others. Women survivors of color are at particular risk of experiencing secondary victimization and institutional betrayal due to intersectional oppression based on their gender, race, type of violence experienced, and, at times, low-income levels (Brown, 2012). Recommendations for reducing secondary victimization and institutional betrayal while improving survivor case engagement include: development of multisystem coordinated care models to reduce survivor burden (e.g., not requiring multiple interviews), improved training for service providers (e.g., sensitivity to trauma), and increased involvement from advocates who can help walk survivors through lengthy and complicated legal processes (Campbell et al., 2001; Long & Garvey, 2012; Murphy et al., 2013). The emerging practice of adult forensic interviews addresses each of these points.

### The Adult Forensic Interview Model

Adult forensic interviews (AFI) are a growing model meant to obtain victim statements and pursue evidence collection in a more trauma-informed way (Hunley & O’Donohue, 2023). Being trauma-informed within organizations and systems means that intentional practices are put in place to minimize the likelihood of survivors experiencing further harm or unnecessary distress (ITTIC, 2022). The first guiding principle of trauma-informed care is to foster safety by designing the spaces which survivors use (e.g., security, aesthetics, accessibility) to help survivors feel physically safe, and designing practices and protocols which encourage practitioners to be attuned to signs of discomfort and help survivors feel emotionally safe (ITTIC, 2022). In their review of AFI protocols for sexual assault, Hunley and O’Donohue (2023) propose AFIs aim to meet four key goals: (a) interviews should not revictimize survivors or discourage continued engagement with law enforcement and the investigatory process; (b) interviewers should be unbiased and collect detailed information to help determine whether or not an assault occurred; (c) interviews should be thorough in their collection of high-quality information for the case, but avoid gathering irrelevant information; and (d) interviewers should be trained in recognizing signs of trauma-related distress in survivors and be prepared to refer to appropriate resources as needed. Implicit throughout these goals is the trauma-informed principle to support survivors’ sense of physical and emotional safety during the evidence collection process; however, whether and how a sense of safety is achieved during AFIs has yet to be evaluated in the literature. Indeed, research on AFI best practices and outcomes, in general, is still in early stages compared to child forensic interviews (Hunley & O’Donohue, 2023; O’Donohue & Fanetti, 2016). Little is known about what survivors think about the AFI process. Only one known study (Kelly & Valentine, 2018) has published data on survivor experiences; among 41 sexual assault survivors who completed this type of interview, 95% reported feeling respected by law enforcement during the investigatory process, with qualitative data indicating they appreciated being able to tell their stories on their own terms in a supportive environment. Notably, the methods and data presented by Kelly and Valentine (2018) are limited as the study assessed AFI-style interviews conducted by law enforcement officers who received only one day of training, and it was unclear where the interviews were conducted (e.g., at a police station vs. an alternate location). Further research on survivor experiences in the context of more well-defined AFI protocols will be a beneficial next step for the field.

### Study Aims

The objective of this study is to explore survivor experiences with and perceptions of AFIs. Specifically, the current study uses mixed methods to explore: (a) aspects of AFI processes which support participants’ sense of safety during the collection of evidence for investigation; and (b) survivor perceptions of utility in completing an AFI.

## Methods

The present study utilizes evaluation data for AFIs conducted by the Adult Forensic Interviewing program (AFI^©^), which serves 12 counties (including both large urban and small rural counties) in Texas. This AFI program is one component of a larger adult forensic center, the Forensic Center of Excellence, which offers medical exams by forensic nurses, interviews conducted by specially trained forensic interviewers, and follow-up advocacy services. The center was established through collaboration between local law enforcement, district attorneys’ offices, hospitals and medical services, and community-based domestic violence/sexual assault advocacy programs. The center is modeled in part after child advocacy centers but is designed to serve survivors aged 17 or older and community-based SANE programs. Through the center, AFI services are available to people who experienced or witnessed interpersonal violence, inclusive of intimate partner violence, family violence, human or labor trafficking, elder abuse, strangulation, sexual assault, or molestation as a child.

### AFI Program for the Current Study

The goals of the AFI program evaluated in the current study align with those established by the broader AFI model (Hunley & O’Donohue, 2023); the program aims to gather information in a sensitive, ethical, and legally defensible manner to support the criminal justice system's response and investigation, while minimizing secondary victimization. To provide context for the transferability and replicability of our findings (Nowell et al., 2017), it is important to provide a detailed description of this specific program. AFIs are set up only through referrals from law enforcement in the partnered counties and typically take place within a week of the reported incident. Two forensic interviewers conduct interviews at a facility outside of law enforcement offices. Tele-forensic interviews are also available using a secure video conferencing platform for participants who are out of state or have barriers to getting to the office. All interviews are video recorded, allowing law enforcement and prosecutors to review survivor statements throughout the investigation without requiring them to sit for additional interviews. The interviewers complete training through a nationally certified and court-recognized forensic interview program and participate in monthly peer reviews to maintain their skills. The interviewers utilize a semi-structured, standardized protocol to support consistency and reliability in evidence collection. The people in the room during AFIs are the interviewer, the survivor, and an interpreter when needed; law enforcement is required to observe all interviews live, either on-site via a closed-circuit camera or off-site via phone or video conference. During AFIs, law enforcement is given an opportunity to provide feedback to the interviewer regarding any additional evidence that may be needed. At the conclusion of AFIs, interviewees are offered the opportunity to meet with an advocate affiliated with the adult forensic center. During fiscal year 2021–2022, the program completed a total of 360 AFIs. The most common type of victimization reported during AFIs was sexual assault (55%), followed by intimate partner violence and/or strangulation (20%), with remaining cases including physical assault, adults molested as children, human trafficking, and kidnapping. Over 40% of AFIs involved more than one type of victimization.

### Participants

To be included in the study, participants had to have completed an AFI with the study program within the previous 12 months and speak either English or Spanish. No other exclusion criteria were considered. Detailed demographic information for participants in each study strand is provided in Table 1.

**Table 1.** Participant Demographics.

	Qualitative interviews^a^ *n* (%)	Quantitative surveys^b^ *n* (%)
Gender		
Woman	13 (87)	35 (92)
Man	2 (13)	2 (5)
Additional gender identity^c^	0 (0)	1 (3)
Age		
18–25	8 (53)	16 (42)
26–45	6 (40)	19 (50)
46 or older	1 (7)	3 (8)
Race/ethnicity		
Hispanic/Latine	8 (53)	14 (37)
Multiracial	3 (20)	3 (8)
Black/African American	2 (13)	11 (29)
White (non-Hispanic)	2 (13)	7 (18)
Asian/Asian American/Pacific Islander	0 (0)	3 (8)
Role in AFI		
Victim of crime	15 (100)	36 (95)
Witness of crime	1 (7)^d^	2 (5)
AFI modality		
In-person	13 (87)	29 (76)
Virtual	2 (13)	9 (24)
Translator present during AFI		
Yes	4 (27)	6 (16)
No	11 (73)	32 (84)

*Note*. ^a^ Sample size = 15; ^b^ Sample size = 38; ^c^ This participant's specific gender identity is withheld due to concerns about anonymity; ^d^ One participant completed an interview about their personal victimization experiences as well as witnessing someone else's experiences.

### Data Collection and Analysis

Data for the current study include 15 qualitative, semi-structured interviews and 38 quantitative online surveys, all completed by recent AFI participants. The interviews were completed first, with initial findings from these data used to inform the development of the online survey. For each data strand, participants were recruited via separate emails sent by the adult forensic center (on behalf of our research team) to anyone who had completed an AFI in the previous 12 months. Any AFI participants who completed an interview were not subsequently invited to participate in the survey. All study protocols were approved by the Institutional Review Board at The University of Texas Medical Branch.

*Qualitative data*. For interviews, participants reached out to our research team directly and set up a time to meet via phone or Zoom. Interviews were conducted in both English and Spanish, lasted 30–60 min, and asked about AFI access, experience, outcomes, and recommendations. Examples of open-ended questions from interviews include: “What was the interview experience like?;” “What did you think about the interviewer?;” and “If you had a friend in a similar situation, would you recommend they participate in an AFI?” Our team made clear that all participant data would remain confidential and that the adult forensic center would not have direct access to their interviews. Participants were provided a $30 gift card following the interview. Interviews were transcribed verbatim using a professional transcription company and uploaded to Atlas.ti software for analysis.

Our team analyzed interview data via inductive thematic analysis, guided by our two research questions (Braun & Clarke, 2021). We began the process by immersing ourselves in the data through readings of the full transcripts. After familiarization, our research team re-read each transcript and individually coded the data to highlight common and unique participant experiences with AFIs. We then met to discuss individual perspectives on the data, began organizing related codes, and developed candidate themes for further investigation. Next, members of our research team reviewed the data again, assessing and refining our candidate themes. The team checked for consistency within each theme and ensured that the themes were distinct from one another. Once we reached consensus on our interpretation and organization of the data, we defined and named themes for the subsequent write-up.

*Quantitative data*. We collected quantitative data via an online survey (hosted on Qualtrics) for the purpose of triangulating our qualitative findings. Most survey questions were self-developed by our research team, drawing on questions and initial findings from the qualitative interviews to assess survivor perceptions of their AFI and potential outcomes post-AFI. Examples of survey questions include, “How long did your forensic interview last?,” with five response options ranging from “0–30 min” to “More than 2 hr.” A follow-up question asked, “The length of my forensic interview was…” with response options: “Too short,” “Too long,” or “Just right.” Participant comfort within the AFI office during interviews was assessed via a 5-point Likert-type scale (ranging from *strongly disagree* to *strongly agree*), which asked whether the office had comfortable temperature, seating, and lighting, and whether survivors felt safe in the space. We also asked participants, “In your opinion, how helpful was the forensic interview for your case?” with 5-point Likert-type responses from *very unhelpful* to *very helpful*. To assess participant perceptions of their AFI interviewer, we adapted the Foundations of Advocacy Behaviors scale (Sullivan et al., 2019), which asks about how much service providers demonstrate key advocacy behaviors using a 4-point Likert-type scale ranging from *not at all* to *very much or a lot*. Full items for our adaptation of this measure can be found in Table 2. As with the interviews, our team made clear during recruitment that data would be confidential and not shared directly with the adult forensic center. Surveys took approximately 15 min to complete and were available in both English and Spanish. Participants received a $20 gift card. We provide descriptive statistics from our survey alongside our related qualitative findings as a form of data triangulation from distinct samples of participants.

**Table 2.** Survivor Perceptions of Adult Forensic Interviewers.

The forensic interviewer…	
Question	*M*
Created a safe environment to share my story	3.86
Explained what was going to happen during the interview	3.68
Asked me if I had any questions	3.79
Listened to me	3.95
Treated me unfairly	1.24
Explained why certain information was needed	3.49
Valued information I shared	3.89
Made me feel pressured to go through with prosecution	1.08
Cared about my unique situation	3.68
Supported me	3.74
Was non-judgmental	3.65
Respected my cultural background and identity	3.97
Said I could take breaks or not answer certain questions	3.76

*Notes.* Sample size = 38 (only survey participants); 4-point Likert-type scale: 1 = *not at all*, 4 = *very much or a lot*.

### Reflexivity and Trustworthiness of Findings

Our authorship team consisted of three faculty researchers, a PhD student, and a community partner from the AFI program. Each of these researchers has extensive field experience working with survivors through direct services and in community-engaged research projects. The community partner provided necessary context on the history, development, and policies and procedures for the program; they were not involved in data collection, analyses, or presentation of our findings. Our collective scholarship aims to support the lives of survivors by improving the services they receive; thus, despite the working relationship with our community partner, our team was intentional in remaining objective.

We aim to bolster the trustworthiness of our findings by providing a clear description of the AFI program being evaluated (i.e., to contextualize transferability) and centering participant voices in our write-up (i.e., to provide confirmability of findings). While recall bias is an inherent threat to retrospective quantitative research, we worked to minimize this amongst our participants by collecting data relatively soon after participating in the AFI (i.e., within one year) and asking clear, neutral questions about their experiences. The credibility of our qualitative findings was supported by researcher triangulation (i.e., independent review and coding of interview data followed by debriefing conversations) as well as data triangulation (i.e., through the development and integration of the quantitative survey data) (Lincoln & Guba, 1985; Nowell et al., 2017).

## Findings

In the current study, we sought to explore: (a) AFI processes which support participants’ sense of safety during the collection of evidence for investigation; and (b) survivor perceptions of utility in completing an AFI. Two primary themes were identified regarding participants’ sense of safety during the interview: (a) creating a safe physical space for interviews (with subthemes: design of the physical space and location); and (b) interviewer techniques (with subthemes: offering a clear description of what to expect; building rapport; attending to participants’ physical needs; having a soothing and respectful demeanor; and allowing survivors to guide the conversation). Regarding the utility of AFIs, we identified three themes related to participants’ perceived benefits to their personal life and case: (a) sharing their story in more detail; (b) Supporting their healing journeys; and (c) feeling safer in the context of their victimization experience. We also provide information on participants’ recommendations (or nuanced reservations) for other survivors to participate in AFIs.

### AFI Practices Supporting Survivors’ Sense of Safety

Participants reflected on several dimensions of AFI policies and procedures, which helped them feel safe and comfortable, ultimately prompting them to share more detailed information about their case with investigators. The practices discussed primarily focused on the physical interview space and techniques used by the interviewers.

#### Creating a Safe Physical Space for Interviews

Participants explained that the *design of the physical space*, described as “like a little living room” (P2), helped them feel both physically and emotionally safe during the interview. Entering a space that felt soothing and nonintimidating was particularly important due to the high levels of anxiety survivors had leading up to the AFIs:

It [the room] felt good. I could feel comfortable, but for an experience like the one I went through, it's like, how can I tell you? I wasn't comfortable to be there, but I felt relieved to be there at the same time. (P1)

For many participants, the anticipatory anxiety over the interview stemmed from lack of information from law enforcement about what the AFI process would look like, “I was actually really scared going up there, because I didn't quite know what to expect. I was envisioning a police station, scary looking” (P14). The *location* of the AFI office, being separate from police stations, reportedly helped survivors feel more comfortable:

It was a really nice environment. Comfy chairs, pillow, blankets if I was cold or wanted one. It was very calming. Green and blue colors in the room. It was like I was in a therapist's office, it didn't feel clinical or scary like the interrogation and interview room at the police station. It was very warm and inviting and calming. It was like safe- it felt like a safe space. (P10)

Another participant shared:

The most important part [of the AFI] for me is, I think just providing the type of environment and the interviewer to clearly tell my side, versus doing it with the police officer or in the police station. It just provides to me a better environment to get comfortable and do that. (P12)

Data from survey participants who completed their AFI in-person supported these findings from the qualitative interviews, with 93% reporting that they felt safe in the AFI office.

#### Interviewer Techniques Which Foster Perceptions of Safety

Participants described a variety of interviewer practices that supported participants’ sense of safety and connection with the interviewer, making them feel comfortable and motivated to share more information about their case. Before initiating the interview, and at times even before entering the room, the interviewers provided a *clear description of what to expect*:

I got to the waiting room area and my interviewer came out […] and in detail told me everything that was gonna happen, everything that it was about. She probably sat there for like 10 or 15 minutes telling me every detail, all of my options before we even went into the interview room. (P10)

Another participant echoed, “It was mostly just her professionalism and her explaining what the process was. I believe that really, really made me more confident on speaking of what happened to me. That really, really helped a lot” (P7). Reflecting the anxiety and misperceptions that many survivors seemed to have going into AFIs, one participant said that how the interview was framed shaped her whole experience: “One thing she said to me, she said, ‘This is an investigation, never interrogation,’ which made me feel a whole lot better about the forensic interview in the first place” (P12).

Key to many of the survivors’ comfort with the AFI interviewers was the time they took to *build rapport* and connect on a human level:

Before we started talking about what had happened, or what we were there for, she asked me if I just wanted to get straight into it or if I wanted to talk to her for a little bit, just to make myself more comfortable. I said I wanted to talk to her. She asked me what school I went to, about what hobbies I had, just stuff like that. She told me her hobbies and stuff, just to get me relaxed. (P14)

Another participant echoed:

I felt like I was very comfortable, and she was very genuine with me. You know how somebody can feel fake and feel like they're just trying to make you feel comfortable but not actually caring about what's being said? It sounds like she was very in it, she cared. (P13)

Part of humanizing and building rapport involved interviewers *attending to the physical needs* of survivors throughout the interview process, including things like providing blankets, snacks, and drinks: “They [the interviewers] made sure that you feel comfortable, you have what you need before they start. They keep checking and making sure that you're comfortable.” (P11). A recurring comment in the qualitative interviews was that the temperature in the AFI office was too cold, to the point that one participant wondered “Is this a tactic? I started to get a little nervous. I was like, why is it so cold?” (P12). The interviewers were responsive to this and tried to accommodate as much as possible:

I noticed how it felt a little bit cold and she was offering to change the AC in the room to try to make it the best environment for feeling as comfortable as possible. […] they offered food and snacks and water and different things to try to calm me down and make me feel safe. (P13)

Nearly all participants spoke highly of how the interviewers conducted their AFIs, describing them as having a *soothing and respectful demeanor*. Survivors were acutely aware of interviewers’ body language and tone of voice and appreciated the neutral responses they provided throughout the interviews. As one participant shared, “What I was scared about was someone judging me, but I felt like she wasn't judging me throughout the whole process, which I really appreciated” (P14). These indicators were also crucial for virtual participants who were not in the physical interview space and only had a close-up view of the interviewer's face to respond to: “She was polite about it. She never made faces or anything. She just allowed me to tell the story […] She told me that she would be taking notes just in case she needed me to clarify something.” (P15, Virtual).

During the evidence collection process, participants also appreciated that the interviewers allowed the *survivors to guide the conversation*, including the pacing and how much they chose to share:

First, she explained to me that due to the situations, it was normal to feel uncomfortable, she told me to relax and to trust. In case I needed time to answer something, to take my time. […]. If anything made me feel anxious or nervous, the same, to take time. If anything made me feel too uncomfortable, it wasn't necessary to mention it, even though it was important. If it was possible for me, to mention it. (P2)

Echoing these sentiments, a virtual participant shared:

She was very nice. She was respectful. She would tell me like, “If you're uncomfortable answering this, that's fine. You totally have the right to not answer it if you don't want to” […] She was okay with me not remembering stuff cause it was so long ago. She wasn't pressing me like, “Well, you said this happened on this day at this time, so why are you saying it happened later then?” She was just real understanding of my situation. (P3, Virtual)

Survivors’ sense of control over the interview was reflected in survey data, which showed that, though there was variability in how long AFIs lasted (ranging from less than 30 min to more than 2 hr, with most falling between 30 and 90 min), 95% of participants stated their interview length was “just right.”

Survey data from our adapted Foundational Advocacy Behaviors scale (Sullivan et al., 2019) supported the generally positive view of the AFI interviewers presented in these qualitative findings (see Table 2). It is important to note, however, that one of the survivors did not perceive the interviewers in the same way as the rest of the participants, stating:

I almost felt like she wasn't on my side actually. It wasn't like it was a conversation with someone who was really caring. It was almost like, “Well, what did you do? Well, why did you do this? Or why did this happen?” Or like, she made me feel like it was something I did to make it happen. (P6)

### Personal and Evidentiary Benefits of AFIs

Survivors perceived the context and environment of the AFI as more conducive to being able to *share their story in more detail* and on their terms, which had purported benefits both personally and for their investigation:

It was the first time I was able to have someone listen and get out the entire story and not feel like any detail was too small. It was the first time for me that I was able to tell the entire story from beginning to end uninterrupted which was very helpful for my own processing. (P10)

Another participant shared:

It helped me to be able to narrate everything comfortably, to be able to digest all that. I do remember that it helped me- I don't know how to express it. It kind of made me feel a little more at ease when narrating all that, compared to the first times when I thought, “No one is going to believe me.” (P2)

Many felt that sharing in this context helped *support their healing journeys* by “getting it off their chests” (P1, P4) and reducing the number of times they must recount the story: “I felt it was helpful to go ahead and get my story recorded […] so I don’t have to tell my story over, and over, and over again” (P6). Some interviewees felt a sense of empowerment after providing evidence to help investigators with their case:

I feel like it did impact me, because I was really- it was mostly my parents that wanted me to do the court and the forensic interview. I was really scared throughout the process […] I did want to tell my story, but at the same time I didn't. Talking to someone like the interviewer, it really helped me be like, “Wow. I can actually talk to people about this.” I should actually be proud of myself for trying to help other people by telling my story. It really did help me throughout the process. (P14)

These benefits extended to those who completed their interviews virtually as well:

But even in the virtual setting, I don't know, I guess having the options to get to know the person or just have it be factual. I think that was pretty much the best part of the process for me. I don't know. It wasn't conversational, but it was just me monologuing basically, but I don't know. Just watching a person listen to me, I guess that was good enough for me. (P15, Virtual)

For several survivors, another benefit was *feeling safer in the context of their victimization experience*. This was primarily related to the provision of resources and specialized knowledge by the center's advocate team:

After the interview, with my case there's some safety concerns…so she talked with me and my mom about things that we could do to keep ourselves safe […] it definitely made me and my mom more aware of some things that we could be doing better or that we just hadn’t thought about. It did make me more comfortable just because she validated that it was not crazy for me to feel like I was unsafe. (P10)

Another survivor stated, “I believe it does make you feel safer. Just in general, the help that they provide does help a lot, actually” (P11). Among survey participants, 82% reported being offered referrals for ongoing support services following their AFI, and 35% reported receiving help addressing their safety needs directly from the center advocate. Others felt safer simply from knowing that law enforcement were taking the time to observe their interviews and that there was now a recording of their experience: “I think that was the first time the detective really saw what I looked like. That was the idea, that she was watching our interview. She could really see- I don’t know- just me as a person” (P8). A Spanish-speaking participant emphasized that, “[The interview] made me feel like I can trust authorities here” (P4).

### Recommendations for Other Survivors to Participate in AFIs

Nearly all interviewees reported feeling positively about their experience with the AFI: “Through this process, I have been thrown into a lot of things. This, by far, was, I felt like the most comfortable and knew the most about what was going to happen” (P10), with most stating they would recommend other survivors provide evidence for their cases in this forum if given the opportunity: “I would recommend it. I would tell them that it may seem scary, but it's not what you think. You’re gonna get there and everyone's going to be welcoming and nonjudgmental” (P14). A participant stated they would encourage others to participate in an AFI by telling them, “You can feel at ease and you can feel that you can say it calmly, without pressure” (P4). Notably, a couple survivors had *nuanced reservations* about recommending AFIs to all survivors: “I would [recommend] if they had a strong enough case […] it's already a traumatic event that happened. And then talking about it before you’re ready is also more traumatic” (P8). Another participant explained:

At this point, I can't really say [if I would recommend] because I haven't had any result…. The investigator is not communicating with me at this point. I would just tell them, yes, go do it, because if you want justice, you're gonna have to press for it, so yes, I could tell them to do it (P6).

This quote may provide context for survey findings, which showed, despite overwhelming positive sentiments about the experience, 66% of survey participants rated their AFI as helpful, while 26% stated they were unsure, and 8% believed it was unhelpful.

## Discussion and Practice Implications

Modeled after child advocacy centers, adult forensic interviews (AFIs) are a newer practice that requires standardization and evaluation (Hunley & O’Donohue, 2023). The objective of the current study was to explore survivor experiences with and perceptions of AFIs, particularly: (a) aspects of AFI processes which support participants’ sense of safety during the collection of evidence for investigation; and (b) survivor perceptions of utility in completing an AFI. The present study extends on previous research (Kelly & Valentine, 2018) to suggest that AFIs are acceptable and considered a positive experience for survivors of sexual assault, IPV, and human trafficking. Notably, this is the first known study to assess AFIs conducted by independent (non-law enforcement), specially trained forensic interviewers in a neutral location (i.e., outside of police stations).

Survivors recounted examples of the AFI structure and procedures that supported their feelings of physical and emotional safety, states that are crucial for conducting successful, trauma-informed interviews about interpersonal violence (Chenier et al., 2021; ITTIC, 2022). Participants generally felt respected, developed a rapport with the interviewer, and felt in control during the AFI. Notably, the diverse sample (with regard to race/ethnicity) of survey participants reported a near-perfect scoring (*M* = 3.97; 4.00 = *very much or a lot*) for the statement, “The forensic interviewer respected my cultural background and identity.” This is a stark contrast to what many women experience when working with violence response services due to the ways that these systems often replicate sociocultural dynamics of sexism, racism, and other dimensions of oppression (Burnett et al., 2018). There are evidentiary benefits for survivors to feel respected and in control during forensic interviews, as research indicates they are likely to share more information if they perceive the interviewer as friendly and can tell their story on their own terms (Gabbert et al., 2020; McMillan & Thomas, 2011). Indeed, participants in our study explained that the AFI context allowed them to recount their victimization experiences in greater detail, which provided more potential corroborating evidence for investigators. Establishing a strong evidence base is crucial in the success of interpersonal violence cases, from convincing prosecutors to file charges, all the way through jury trials and judges’ sentencing (Brown, 2012; Spohn & Tellis, 2019). While maintaining survivor participation can be crucial to the ultimate success of these cases, a detailed AFI can provide guidance for evidence-based prosecution strategies, even in situations where a survivor disengages (Long & Garvey, 2012; Messing, 2014).

Based on our findings, AFIs seem to have potential for reducing secondary victimization and experiences of institutional betrayal during the investigatory process, which may help restore trust in parts of the criminal legal system. Research demonstrates there can be psychological benefits to survivors participating in criminal legal processes (e.g., by improving self-esteem and decreasing avoidance coping; Parsons & Bergin, 2010), findings which were echoed by our participants who felt the AFI supported (rather than harmed) their healing journeys. Moreover, many participants indicated they would promote other survivors to report and participate in an AFI. This is an encouraging finding given that secondary institutional betrayal (hearing negative accounts of other peoples’ help-seeking experiences) can discourage survivors from reporting and accessing services (PettyJohn, Kynn, et al., 2023). A potential caveat to this is that AFIs are likely only supportive of survivor experiences so far as there is continued communication and coordination of services with law enforcement and prosecutors afterwards. Despite widely positive personal experiences, a quarter of participants stated they were “unsure” whether the AFI was helpful in their legal case, a sentiment likely tied to a lack of follow-up communication after the interview.

Importantly, our study included survivors who completed virtual AFIs, and these participants reported similarly positive experiences to survivors who completed their AFIs in person. This is a notable finding in the context of broader efforts to make forensic medical care and response services more accessible for victims of interpersonal violence via telehealth technologies. The nursing field has created practice models for conducting sexual assault forensic exams via telehealth (SAFE-T; Miyamoto et al., 2021), with preliminary evaluation data demonstrating success in improving survivor care in settings that did not have an in-person SANE available (Miyamoto et al., 2022). Further research on virtual AFIs is warranted, as the ability to offer this service in conjunction with teleforensic exams could help address disparities in trauma-informed survivor services and strengthen evidence collection for crimes of interpersonal violence in rural and underserved communities.

### Limitations and Future Research

Our findings suggest that the AFI program being evaluated was successful in meeting some of the goals of AFIs proposed by Hunley & O’Donohue (2023), namely that interviews should not revictimize survivors and that interviewers should be responsive to trauma-related distress throughout the process. However, the evaluation and resultant impacts of Hunley & O’Donohue's (2023) other goals for AFIs (i.e., continued engagement with the investigation; collection of unbiased, high-quality evidence for prosecution) were not assessed and should be explored in future work. Data for the current study were collected relatively soon after AFI participation (within the previous year), which did not provide much time to assess continued case participation among survivors or outcomes from criminal legal systems. Likewise, we did not have the necessary time to assess how the evidence collected during the AFIs impacted prosecutorial processes and outcomes. Echoing critiques from research on CACs (Herbert & Bromfield, 2016), our study operated from certain assumptions; namely, that AFI participants who feel safe during their interviews will experience lower rates of secondary victimization and be more likely to remain engaged in criminal legal processes. However, these constructs need to be more systematically measured through longitudinal follow-ups and comparison to controls who did not participate in an AFI as part of their investigatory process. Our sample consisted predominantly of women. This is an exploratory and novel study; therefore, expanded research on AFI models with women is needed, as is work examining AFI models across gender and age, as these communities have distinctly different experiences with the criminal legal system (Miles-Johnson, 2020; PettyJohn, Reid, et al., 2023). Process research on how AFIs are used by the criminal legal system, specifically assessment of whether they reduce burden on system actors, improve documentation, or increase corroborating evidence, is also needed.

## Conclusion

Adult forensic interviewing (AFI), modeled after child advocacy centers, is an emerging practice that seeks to collect thorough evidence for investigating interpersonal violence crimes while using trauma-informed techniques to support the well-being of survivors. Findings from our study suggest the physical design of the interview space (e.g., outside of police stations) and certain interviewer techniques (e.g., allowing survivors to guide the conversation) support a sense of safety among participants and result in the collection of more detailed evidence to bolster ongoing investigations. Survivors in the present study felt that completing an AFI supported their healing journeys in various ways, and most stated they would encourage others to participate if given the chance. AFIs are a promising practice with potential for improving survivors’ experiences with criminal legal systems and increasing successful prosecution of interpersonal violence cases; however, further implementation and evaluation of the AFI model is needed.
